# A comparison of methods to estimate the survivor average causal effect in the presence of missing data: a simulation study

**DOI:** 10.1186/s12874-019-0874-x

**Published:** 2019-12-03

**Authors:** Myra B. McGuinness, Jessica Kasza, Amalia Karahalios, Robyn H. Guymer, Robert P. Finger, Julie A. Simpson

**Affiliations:** 1 Centre for Eye Research Australia, Royal Victorian Eye and Ear Hospital, Melbourne, Australia; 2 Centre for Epidemiology and Biostatistics, Melbourne School of Population and Global Health, University of Melbourne, Melbourne, Australia; 3 Department of Epidemiology and Preventive Medicine, Monash University, Melbourne, Victoria 3010 Australia; 4 Ophthalmology, Department of Surgery, University of Melbourne, Melbourne, Australia; 5 Department of Ophthalmology, University of Bonn, Bonn, Germany; 6 Cancer Epidemiology Centre, Cancer Council Victoria, Melbourne, Australia

**Keywords:** Causal inference, Death, Iron, Macular degeneration, Missing data, Principal stratification, Sensitivity analysis, Simulation study, Survival bias, Unmeasured confounding

## Abstract

**Background:**

Attrition due to death and non-attendance are common sources of bias in studies of age-related diseases. A simulation study is presented to compare two methods for estimating the survivor average causal effect (SACE) of a binary exposure (sex-specific dietary iron intake) on a binary outcome (age-related macular degeneration, AMD) in this setting.

**Methods:**

A dataset of 10,000 participants was simulated 1200 times under each scenario with outcome data missing dependent on measured and unmeasured covariates and survival. Scenarios differed by the magnitude and direction of effect of an unmeasured confounder on both survival and the outcome, and whether participants who died following a protective exposure would also die if they had not received the exposure (validity of the monotonicity assumption). The performance of a marginal structural model (MSM, weighting for exposure, survival and missing data) was compared to a sensitivity approach for estimating the SACE. As an illustrative example, the SACE of iron intake on AMD was estimated using data from 39,918 participants of the Melbourne Collaborative Cohort Study.

**Results:**

The MSM approach tended to underestimate the true magnitude of effect when the unmeasured confounder had opposing directions of effect on survival and the outcome. Overestimation was observed when the unmeasured confounder had the same direction of effect on survival and the outcome. Violation of the monotonicity assumption did not increase bias. The estimates were similar between the MSM approach and the sensitivity approach assessed at the sensitivity parameter of 1 (assuming no survival bias). In the illustrative example, high iron intake was found to be protective of AMD (adjusted OR 0.57, 95% CI 0.40–0.82) using complete case analysis via traditional logistic regression. The adjusted SACE odds ratio did not differ substantially from the complete case estimate, ranging from 0.54 to 0.58 for each of the SACE methods.

**Conclusions:**

On average, MSMs with weighting for exposure, missing data and survival produced biased estimates of the SACE in the presence of an unmeasured survival-outcome confounder. The direction and magnitude of effect of unmeasured survival-outcome confounders should be considered when assessing exposure-outcome associations in the presence of attrition due to death.

## Background

Attrition due to death and loss to follow-up are two major potential sources of bias in observational studies which investigate diseases of ageing. Statistical methods have been proposed to estimate exposure-outcome effects in the presence of each attrition scenario separately, however little is known about how the methods compare when both sources of attrition are present.

As an illustrative example, we examine the causal effect of dietary iron intake on age-related macular degeneration (AMD). Intracellular iron has been implicated in the pathogenesis of several chronic diseases of ageing, including AMD [1–3]. AMD is a chronic eye disorder responsible for severe and irreversible visual impairment in older adults. Despite evidence of elevated levels of iron in the retinal tissue of individuals with AMD, there is little evidence to suggest a link between dietary iron intake levels and the development of AMD [4].

As observed when investigating associations between other lifestyle factors and AMD, quantification of the effect of iron intake on AMD is susceptible to survival bias [5]. Individuals at risk of AMD also face the competing risk of death, and loss to follow-up is also common among elderly cohorts for whom ill-health and poor mobility can hinder attendance at study visits. When survival is associated with the exposure of interest and only participants who survive until the outcome wave are included in an analysis, exposure groups may lose exchangeability. Exchangeability implies that, conditional on the observed characteristics of individuals, an estimate of the causal relationship between the exposure and the outcome can be obtained. That is, balance of confounding variables is achieved across each category of the exposure. A loss of exchangeability can occur when there are shared predictors of survival and the outcome; in this case bias may be observed regardless of statistical adjustment for all direct exposure-outcome confounders.

The survivor average causal effect (SACE) has been proposed as a parameter to assess exposure-outcome relationships in analyses that are susceptible to survival bias. The SACE exists within the potential outcomes framework, which requires us to consider all participants’ potential outcomes under each level of the exposure, and uses principal strata categorising the potential survival of each subject under each level of exposure to define the relevant causal effect [6]. The SACE is a measure of the average causal effect of the exposure on the outcome among participants who would survive regardless of their exposure status, commonly referred to as always-survivors [7]. Over the last two decades several methods have been developed to estimate the SACE, each requiring various assumptions to ensure identifiability [7–10].

Marginal structural models (MSMs) have been employed by several authors to estimate causal effects (including the SACE) and have been extended to account for participants with missing outcome data due to non-attendance [11–15]. Shardell and co-authors have shown that inverse probability weighting for survival can produce unbiased estimates for the SACE when survival has been correctly modelled and there are no unmeasured survival-outcome confounders [13]. Another MSM approach proposed by Tchetgen Tchetgen involves weighting for the probability of being an always-survivor [14]. Other methods to estimate the SACE make assumptions regarding the potential outcomes of surviving participants, including those with missing outcome data, without explicitly modelling the distribution of missing outcome data. For these methods, missing outcome data for surviving participants are considered to be missing at random and potential outcomes can be generated for all baseline participants regardless of their attendance at the follow-up [16]. An example of this is a sensitivity analysis approach proposed by Egleston and co-authors [17].

In this paper we present a simulation study to compare the performance of two methods employed to estimate the SACE in the presence of missing binary outcome data due to death and loss to follow-up. One method involves a MSM (employing inverse probability weights for exposure, survival and loss to follow-up) and the other method employs the sensitivity analysis approach described above. These approaches have been chosen as they have previously been demonstrated in studies with binary outcomes.

As an illustrative example we use data from the Melbourne Collaborative Cohort Study to estimate the association between iron intake (for simplicity expressed as binary exposure measured at baseline; low vs high sex-specific iron intake) and the presence of the late stage of AMD at a follow-up study wave.

## Methods

### Notation and framework for potential outcomes

For each participant, *i*, let an indicator of the observed exposure at baseline, *A*_*i*_, equal 0 for low iron intake and 1 for high iron intake. Let *Z*_*i*_ = 1 if participant *i* is alive at the start of the follow-up wave, and *Z*_*i*_ = 0 otherwise. Let the outcome, *Y*_*i*_, equal 1 in the presence of AMD at the follow-up wave and 0 otherwise. For participants who have died before the start of the follow-up wave, *Y*_*i*_ is undefined. *R*_*i*_ is an indicator of attendance at the follow-up wave for participant *i*; again it is undefined for participants who die before that wave. Participant characteristics, (such as age and sex, represented by the vector **V**) are collected at baseline. *U*_*i*_ is an indicator of a genotype which is associated with both general health (and, therefore, survival) and the outcome, AMD. In the simulation study below *U*_*i*_ represents an unmeasured variable. *D*_*i*_ is an indicator for location of residence; with participants living in lower socio-economic status areas less likely to attend the follow-up wave. In the simulation study below, *D*_*i*_ also represents an unmeasured variable. There are *N* participants at the baseline wave with *n*_*A* = 0_ participants observed to have low iron intake, *n*_*A* = 1_ participants observed to have high iron intake (*N* = *n*_*A* = 0_ + *n*_*A* = 1_ ). *n*_*Z* = 1_ participants are observed to survive until the follow-up study wave.

**Fig. 1:**
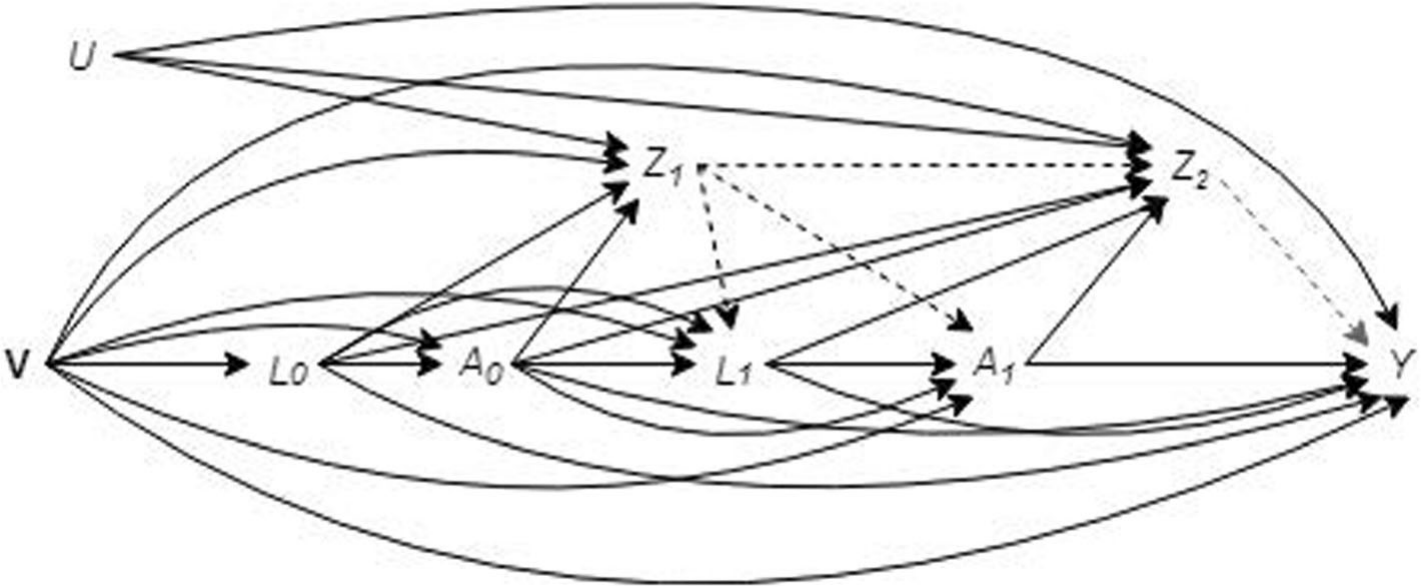
Causal diagram for the effect of iron intake on age-related macular degeneration. V represents the vector of participant demographics (e.g. age and sex) recorded at baseline. Exposure, *A*, is also recorded at baseline. *Z* is an indicator of survival until the start of the follow-up wave. *R* is an indicator of attendance at the follow-up study wave when outcome (*Y*, age-related macular degeneration) was ascertained. An indicator genotype, *U*, is unmeasured, as is *D*, an indicator for area of residence. **a** A scenario where missing outcome data are missing at random. **b** Conditioning on *Z* (a collider between the exposure and *U*) will unblock the backdoor pathway (dashed line) from the exposure to the outcome through *U*

As seen in the causal diagram presented in Fig. 1a, survival (*Z*) is a collider variable on the pathway between the exposure (*A*) and the outcome (*Y*) since it is a “child” of the exposure and genotype (*U*) [18]. Participant inclusion in the analysis is dependent on survival until the follow-up wave. Conditioning on survival unblocks the backdoor pathway between the exposure and the outcome through *U* (as seen in Fig. 1b) which introduces confounding bias [19].

Under the framework of potential outcomes we can consider an individual’s outcomes if we were to set the exposure (iron intake) to *a*, where *a* can take on the values 0 (low iron intake) or 1 (high iron intake) [6]. When the value of the potential exposure for individual *i* (*a*_*i*_) is contrary to their observed exposure (*A*_*i*_) the potential outcome under that potential exposure is often referred to as the counterfactual outcome [20]. *Z*_*i*_(*a*) is the potential survival status and *Y*_*i*_(*a*) is the potential AMD status for participant *i* when iron intake is set to *a*.

Principal stratification refers to categorization of individuals according to their potential survival outcome for each level of iron intake [21]. The first stratum consists of always-survivors, the individuals who are the most robust and will be alive at follow-up regardless of iron intake level (*Z*_*i*_(*a* = 0) = *Z*_*i*_(*a* = 1) = 1). The next stratum is comprised of never-survivors, those who are the most fragile and will not survive irrespective of exposure level (*Z*_*i*_(*a* = 0) = *Z*_*i*_(*a* = 1) = 0). Compliant-survivors will survive only if they have high iron intake (*Z*_*i*_(*a* = 0) = 0, *Z*_*i*_(*a* = 1) = 1), whereas defiant-survivors will survive only if they have low iron intake (*Z*_*i*_(*a* = 0) = 1, *Z*_*i*_(*a* = 1) = 0).

Principal stratum does not change after a participant has been exposed and therefore is considered to be a pre-exposure variable. A person’s principal stratum will depend on complex interactions between genetics, past behaviour and environmental factors, many of which are unlikely to be measureable. In the simulation study below, these factors are represented by the variable *U* which is also a predictor of the outcome. Predictors of survival determine which participants are compliant-survivors. After conditioning on survival (by including data from surviving participants only), the distribution of these variables become unbalanced between principal strata and, therefore, exposure groups; surviving participants with higher levels of the exposure, iron intake, (who include always-survivors and compliant-survivors) will have differing levels of *U* compared to surviving participants with lower levels of iron intake (who are all always-survivors). To obtain an unbiased estimate of the exposure-outcome effect, predictors of the outcome which are not evenly distributed between exposure groups (high vs low iron intake) must be accounted for in the analysis. However, traditional statistical methods cannot adjust for variables, such as *U*, that have not been measured. Therefore, survival bias exists when confounding of the relationship between exposure (iron intake) and the outcome (AMD) by *U* is induced after conditioning on survival.

**Fig. 2:**
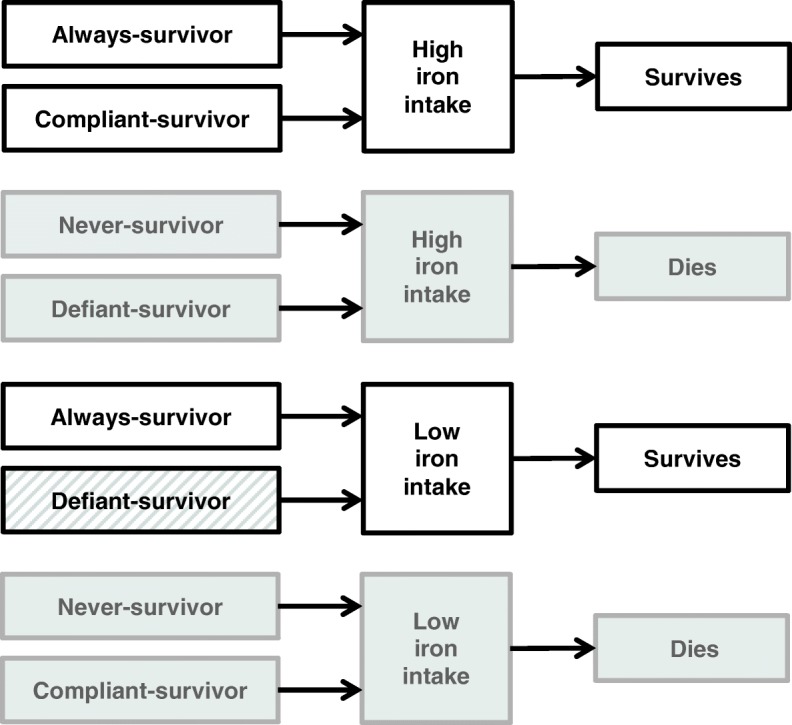
Identification of principal strata dependant on observed exposure and survival status. Under the assumption of monotonicity, there are no defiant-survivors; all survivors who had low iron intake at baseline can be identified as always-survivors but survivors who had high iron intake at baseline could be always-survivors or compliant-survivors. When the monotonicity assumption has been violated, participants who survive following low iron intake may be always-survivors or defiant-survivors (grey stripes). White boxes represent survivors and grey boxes represent those who do not survive

One assumption commonly required to identify the SACE is monotonicity. The monotonicity assumption states that iron intake will have a non-negative effect on survival. That is, participants who survive with low iron intake will also survive with high iron intake, and participants who die with high iron intake will not survive with low iron intake (*Z*_*i*_(*a* = 0) ≤ *Z*_*i*_(*a* = 1)). Under the assumption of monotonicity, there will be no defiant-survivors. Survivors who have low iron intake must be always-survivors; whereas survivors with high iron intake could be either always-survivors or compliant-survivors (as seen in Fig. 2). Consequently, individuals with low iron intake are not directly comparable to (or exchangeable with) those with high iron intake at the outcome study wave.

### The survivor average causal effect (SACE)

Under the assumption of no unmeasured confounders between the exposure and outcome (sometimes referred to as the strong ignorability assumption) and the assumption that principal stratum is a pre-exposure variable, the exposure groups will be exchangeable when analyses are restricted to the stratum of always-survivors [22]. Hence, there is no survival bias when assessing the association between the exposure (iron intake) and the outcome (AMD) among this subgroup of participants. Therefore, the SACE odds ratio (*SACE*_*OR*_) is defined as the ratio of the odds of AMD when *a* = 1 (high iron intake) to the odds of AMD when *a* = 0 (low-iron intake) among always-survivors (*AS*):

1 $$ {\boldsymbol{SACE}}_{\boldsymbol{OR}}=\frac{\mathrm{odds}\left[\boldsymbol{Y}\left(\boldsymbol{a}=\mathbf{1}\right)=\mathbf{1}|\boldsymbol{AS}\right]}{\mathrm{odds}\left[\mathbf{Y}\left(\boldsymbol{a}=\mathbf{0}\right)=\mathbf{1}|\boldsymbol{AS}\right]} $$

However, as seen in Fig. 2, it is not possible to identify which participants are always-survivors without additional assumptions such as those described below.

The SACE is a marginal effect, meaning that it aims to reflect the effect of an exposure on an outcome averaged over the levels of unmeasured confounders within a specified population. This is in contrast to the conditional odds ratio estimated via traditional logistic regression with covariate adjustment. The conditional odds ratio will coincide with the SACE when there is no survival bias and the covariates do not modify the effect of the exposure on survival. However, it should be noted that, unlike mean differences, risk differences or risk ratios, odds ratios are non-collapsible, meaning that the unadjusted odds ratio for the entire sample cannot be expressed as a weighted average of odds ratios from each observed pattern of confounders. Therefore, when estimating odds ratios, conditional effects may differ from marginal effects even in the absence of confounding [23].

### Estimation of the survivor average causal effect

Many approaches have been proposed to estimate the SACE [24–29]. The approaches under comparison in this study are described below. Example statistical computing code for estimating the SACE via these methods in Stata is available in the Additional file 1. The approaches presented below require the assumption that the outcome data are missing conditional on measured covariates, and that missingness is independent of the outcome [30]. In addition, each of the methods invoke the stable unit treatment value assumption, which states that the outcome of one participant is not dependent on the exposure of another, and that there is only one version of the exposure (i.e. the level of exposure is the same for everyone who has been categorised as having high iron intake) [31]. It is also assumed that it is possible for participants to have been exposed to either exposure level (i.e. low and high iron intake) for every observed pattern of exposure-outcome confounders; this is known as the positivity assumption [32].

#### Estimating marginal structural models (MSMs) with standardised weights for survival

This approach is similar to that presented by Shardell and co-authors in 2015 [13]. It uses stabilised inverse probability of observed exposure weights to achieve balance of measured baseline covariates across exposure groups.

The analysis of the exposure-outcome association is conducted via weighted logistic regression, whereby each participant, *i*, is weighted by an estimate of *W*_*i*_:

2 $$ {\boldsymbol{W}}_{\boldsymbol{i}}=\frac{{\boldsymbol{SF}}_{\boldsymbol{A}={\boldsymbol{A}}_{\boldsymbol{i}}}}{\left({\boldsymbol{A}}_{\boldsymbol{i}}{\boldsymbol{p}}_{\boldsymbol{i}}+\left(\mathbf{1}-{\boldsymbol{A}}_{\boldsymbol{i}}\right)\left(\mathbf{1}-{\boldsymbol{p}}_{\boldsymbol{i}}\right)\right){\boldsymbol{q}}_{\boldsymbol{i}}{\boldsymbol{m}}_{\boldsymbol{i}}} $$

where *i* = 1, 2, …, *N* and *A*_*i*_ = 0, 1. Here, *p*_*i*_ is the propensity for having high iron intake for the *i* ^th^ participant (Eq. 3). The propensity is estimated via logistic regression adjusted for all measured exposure-outcome confounders and strong predictors of the outcome, represented by the vector **P** [33]:.

3 $$ \Pr \left[{\boldsymbol{A}}_{\boldsymbol{i}}=\mathbf{1}\ |\boldsymbol{P}={\boldsymbol{P}}_{\boldsymbol{i}}\right]={\boldsymbol{p}}_{\boldsymbol{i}} $$

*q*_*i*_ is the propensity for survival until the follow-up study wave for the *i* ^th^ participant (Eq. 4). It is estimated via logistic regression adjusted for the exposure (iron intake), all measured survival-outcome (AMD) confounders and strong predictors of the outcome. These covariates are represented here by the vector **Q**. Because *U* represents an unmeasured variable, it cannot be included as a covariate when estimating the probability of survival:

4 $$ \Pr \left[{\boldsymbol{Z}}_{\boldsymbol{i}}=\mathbf{1}\ |\boldsymbol{A}={\boldsymbol{A}}_{\boldsymbol{i}},\mathrm{Q}={\mathrm{Q}}_{\boldsymbol{i}}\right]={\boldsymbol{q}}_{\boldsymbol{i}}; $$

*m*_*i*_ is the propensity for attendance at the follow-up wave among the participants who survive (Eq. 5). This propensity is estimated via logistic regression adjusting for iron intake, all measured attendance-outcome confounders and strong predictors of the outcome. These covariates are denoted by the vector **M**. Information on area of residence is unmeasured and therefore *D* is not included as a model covariate in this example:

5 $$ \Pr \left[{\boldsymbol{R}}_{\boldsymbol{i}}=\mathbf{1}\ |{\boldsymbol{Z}}_{\boldsymbol{i}}=\mathbf{1},\boldsymbol{A}={\boldsymbol{A}}_{\boldsymbol{i}},\mathrm{M}={\mathrm{M}}_{\boldsymbol{i}}\right]={\boldsymbol{m}}_{\boldsymbol{i}}; $$

*SF*_*A*_ is a stabilising factor used to improve the efficiency of the weights (Eq. 6) [11]. Here, it is the average of the propensity for each exposure level. Separate constant values are used as stabilising factors for participants observed to have low iron intake (*SF*_*A* = 0_) and participants observed to have high iron intake (*SF*_*A* = 1_) [34].

6 $$ {\boldsymbol{SF}}_{\boldsymbol{A}}=\frac{\mathbf{1}}{{\boldsymbol{n}}_{\boldsymbol{A}={\boldsymbol{A}}_{\boldsymbol{i}}}}\left(\sum \limits_{\boldsymbol{i}=\mathbf{1}}^{{\boldsymbol{n}}_{\boldsymbol{A}={\boldsymbol{A}}_{\boldsymbol{i}}}}{\boldsymbol{p}}_{\boldsymbol{i}}\right) $$

*W*_*i*_ is undefined for participants who do not survive until the follow-up study wave because non-missing outcome status can only be defined for survivors. As with any method which utilises propensity scores, the balance of covariates across exposure groups should be assessed before and after applying weights [33].

Finally a weighted logistic regression is fitted to the binary outcome (*Y*) with the covariates, the exposure variable (*A*) and baseline confounders (**V**). In the presence of stabilised inverse probability weights, further adjustment for baseline confounders of the exposure-outcome relationship in the final weighted logistic regression can reduce bias [35]. The regression coefficient for the exposure is taken as the estimate of the log-odds of the SACE for this approach.

By incorporating the probability of attendance at the follow-up wave in the weight, participants with observed outcomes represent those with similar characteristics who were lost to follow-up. Weighting for survival plays a similar role; the surviving participants with the lowest propensity for survival are given more weight to represent those participants with similar characteristics who have died.

#### Estimation for sensitivity analysis approach

Survivors who reported high iron intake at baseline, i.e. when *A* = 1, could be always-survivors or compliant-survivors (see Fig. 2); the strata to which they belong is not directly identifiable from examination of the data. A sensitivity analysis approach was proposed by Egleston and co-authors in 2007 to reflect this uncertainty [17]. In this approach, the sensitivity parameter (*τ*) represents the ratio of the odds of AMD among the compliant-survivors (*CS*) to the odds of AMD among the always-survivors (*AS*) when *a* is set to one:

7 $$ \boldsymbol{\tau} =\frac{\mathrm{odds}\left[\boldsymbol{Y}\left(\boldsymbol{a}=\mathbf{1}\right)=\mathbf{1}|\boldsymbol{CS}\right]}{\mathrm{odds}\left[\boldsymbol{Y}\left(\boldsymbol{a}=\mathbf{1}\right)=\mathbf{1}|\boldsymbol{AS}\right]} $$

As indicated in the original paper, *τ* is a marginal value and it is assumed to be constant over all values of the baseline covariates [17]. If individuals with higher values of *U* are less likely to survive and have a higher probability of AMD then it is assumed that always-survivors will be more robust and less likely to develop AMD than those who are compliant-survivors. In this scenario, the value of *τ* will be greater than one. However, if higher values of *U* are associated with a lower probability of both survival and AMD, then the value of *τ* will be less than one. Likewise, when *U* is associated with a higher probability of both survival and AMD, the value of *τ* will also be less than one.

As described in the 2007 paper, when *τ* is not equal to one the *SACE*_*OR*_ is equivalent to [17]:.

8 $$ {\displaystyle \begin{array}{c} SAC{E}_{OR}\left(\tau \ne 1\right)=\frac{\left({\nu}_0+{\xi}_1\right)\left(\tau -1\right)-\tau {\nu}_1+q}{\left({\nu}_0-{\xi}_1\right)\left(\tau -1\right)+\tau {\nu}_1-q}\times \frac{\nu_0-{\xi}_0}{\xi_0}\\ {}\mathrm{where}\ q=\sqrt{{\left\{\left({\nu}_0+{\xi}_1\right)\left(1-\tau \right)+\tau {\nu}_1\right\}}^2+4{\xi}_1{\nu}_0\left(\tau -1\right)}\end{array}} $$

Here, *ν*_*a*_ is the marginal probability of survival and *ξ*_*a*_ is the marginal probability of both surviving and having AMD when iron intake has been set to *a*. As the value of *τ* is not identifiable from the data, a sensitivity analysis can be conducted over a range of values for *τ*. Content matter experts must decide which values of *τ* are plausible in the context of the analysis.

To estimate the value of *ξ*_*a*_, the predicted probability of the outcome must first be estimated via covariate adjusted logistic regression, separately for participants observed to have *A* = 0 (low iron intake) and for participants observed to have *A* = 1 (high iron intake). All measured predictors of AMD are included as covariates. The coefficients from these models are then used to predict the probability of AMD, *h*_*i*_(*a*), for all surviving participants (regardless of missing outcome data status) under both observed and counterfactual levels of the exposure:

9 $$ {\displaystyle \begin{array}{c}\mathit{\Pr}\left[{Y}_i(a)=1\ |V={V}_i,{Z}_i=1\right]={h}_i(a)\\ {}\mathrm{where}\;i=1,2,\dots, {n}_{Z=1}\;\mathrm{and}\;a=0,1\end{array}} $$

The adjusted potential probability of survival under each exposure, *g*_*i*_(*a*), is also estimated for each participant:

10 $$ {\displaystyle \begin{array}{c}\mathit{\Pr}\left[{Z}_i(a)=1\ |Q={Q}_i\right]={g}_i(a)\\ {}\mathrm{where}\;i=1,2,\dots, N\;\mathrm{and}\;a=0,1\end{array}} $$

Under each potential exposure level, the predicted probability of AMD (*h*_*i*_(*a*), Eq. 9) is multiplied by the predicted probability of survival (*g*_*i*_(*a*), Eq. 10) for each surviving participant. The average is then taken as the estimate for *ξ*(*a* = 0) or *ξ*(*a* = 1), i.e. the marginal probability of surviving and having AMD under low or high iron intake, respectively:

11 $$ {\displaystyle \begin{array}{c}{\xi}_a=\frac{1}{n_{Z=1}}\sum \limits_{i=1}^{n_{Z=1}}{g}_i(a){h}_i(a)\\ {}\mathrm{where}\kern0em i=1,2,\dots, {n}_{Z=1}\;\mathrm{and}\;a=0,1\end{array}} $$

Under the assumption of monotonicity, when *τ* is equal to 1 the *SACE*_*OR*_ is equivalent to:

12 $$ {\boldsymbol{SACE}}_{\boldsymbol{OR}}\left(\boldsymbol{\tau} =\mathbf{1}\right)=\frac{{\boldsymbol{\xi}}_{\mathbf{1}}\left({\boldsymbol{\nu}}_{\mathbf{0}}-{\boldsymbol{\xi}}_{\mathbf{0}}\right)}{{\boldsymbol{\xi}}_{\mathbf{0}}\left({\boldsymbol{\nu}}_{\mathbf{1}}-{\boldsymbol{\xi}}_{\mathbf{1}}\right)} $$

The proof for this equation is given in the Additional file 1. Note that, under monotonicity, when *τ* = 1 the distribution of the outcome is equal between always- and compliant-survivors, and no survival bias is thought to be present. However, the marginal estimate derived from Eq. 12 may be different to the traditional conditional estimate if measured covariates modify the effect of the exposure on survival [36].

### Simulation study

#### Data generation

A dataset of 10,000 individuals was generated to allow substantial proportions of death and missing data while still observing a relatively rare outcome within subgroups of participants. Full details of the data generation process including model parameters and a flowchart are provided in the Additional file 1.

Binary variables for sex (*V*1), genotype (*U*) and location of residence (*D*) were generated randomly. Mean-centred age (*V*2) was generated under a uniform distribution. The exposure of interest, a binary indicator of iron intake (*A*), was then generated conditional on sex and age. The propensity for survival was generated conditional on the exposure, sex, age and genotype. The value of the coefficient for genotype varied between scenarios (*α*_*UZ*_ =   ln(0.5) or ln(2)). This propensity was then used to generate a binary indicator for survival under potential exposure to high iron intake (*Z*(*a* = 1)) for every participant. For scenarios generated to be compliant with the monotonicity assumption, potential survival under low iron intake (*Z*(*a* = 0)) was generated among those who would survive under high iron intake (i.e. for those with *Z*_*i*_(*a* = 1) = 1). For scenarios generated in violation of the monotonicity assumption, *Z*(*a* = 0) was generated for all participants regardless of the value of *Z*_*i*_(*a* = 1). Survival status (*Z*) was then assigned deterministically according to exposure (*A*_*i*_) and potential survival outcome *Z*_*i*_(*a* = *A*_*i*_). Principal strata (created due to the presence of the unmeasured confounder which is a pre-exposure variable) were then identified according to the potential survival outcome under each exposure level as described above.

Potential outcome variables were generated for each exposure level (*Y*(*a* = 0) for low iron intake and *Y*(*a* = 1) for high iron intake) dependent on sex, age and genotype for participants who would survive under that exposure level. The value of the coefficients for genotype (*β*_*UY*_ = ln(0.5), ln(1) or ln(2)) varied between scenarios. Because survival (and therefore principal strata) is also dependent on genotype, the distribution of the outcome will differ between always-survivors and compliant-survivors. The marginal odds ratio for the difference between *Y*(*a* = 1) and *Y*(*a* = 0) was set to 0.6. This is the true value of the SACE and was chosen to reflect the estimated direction and magnitude of effect of iron on AMD in the illustrative example.

An indicator of attendance at follow-up (*R*) was generated conditional on *V*1, *V*2, *A* and *D* among surviving participants (i.e. when *Z* = 1).

The observed value of *Y* (AMD) was then assigned the value of *Y*(*a* = 0) or *Y*(*a* = 1) deterministically depending on the allocated exposure (*A* = 0 or *A* = 1 respectively), survival status (*Z*) and attendance status (*R*). A total of 1200 datasets were generated for each scenario. The combinations of parameters for each of the 12 scenarios are given in of the Additional file 1: Table S2 .

#### Simulation study analysis methods

Four estimates were recorded for each of the generated datasets: (1) the log-odds of the SACE estimated via a MSM using standardised inverse probability weights for the probability of observed exposure, survival and non-attendance; the sensitivity analysis approach, (2) evaluated using a sensitivity parameter of 1 (Eq. 12); (3) a sensitivity parameter of 0.5 and (4) a sensitivity parameter of 2 (Eq. 8).

The SACE and value of *τ* were estimated for each dataset using the known values of the potential outcomes (*Y*(*a* = 0) and *Y*(*a* = 1)) and principal strata. The empirical value of these parameters for each scenario was then determined by averaging the estimates derived from the 1200 generated datasets.

The absolute bias for each method was calculated within each scenario as the difference between the true SACE (odds ratio = 0.6) and the average parameter estimate (on the log-odds ratio scale) calculated across 1200 simulated datasets. The empirical standard error for each method was calculated as the standard deviation of the estimates from all simulations in each scenario [37]. To assess accuracy, the absolute bias and the empirical standard error were used to calculate the mean square error (MSE) for each estimation method under each scenario. Standardised bias was calculated as a percentage of the absolute bias relative to the empirical standard error. Bias-corrected 95% confidence intervals were generated via 1000 bootstrap samples for the MSM estimate of each dataset generated and coverage was estimated as the percentage of datasets in which the confidence interval included the true value of the SACE within each scenario [38].

### Example dataset

The Melbourne Collaborative Cohort Study is a prospective community-based study of 41,514 people living in Melbourne, Australia. Details of the study have been published elsewhere [39, 40]. In brief, participants attended baseline clinics (1990–1994) where information on demographics, lifestyle and diet was collected. Colour digital fundus photography was performed a median of 11.8 years after baseline attendance, between 2003 and 2007.

The study protocol was approved by the Human Research and Ethics Committees of The Cancer Council Victoria and the Royal Victorian Eye and Ear Hospital, and was conducted in accordance with the Declaration of Helsinki. Written informed consent was obtained from all participants after explanation of the nature of the study.

Iron intake over the year prior to attendance at the baseline study wave was estimated using a 121-item food frequency questionnaire. Iron content in food and beverages (excluding supplements) was derived from Australian food composition tables (NUTTAB 1995) [41]. Participants were considered to have high iron intake if their intake was above the median for their sex. Participants with iron intake below the 1st and above the 99th sex-specific centiles of the baseline population were considered to have potential measurement error and were excluded.

Sex, age, country of birth, smoking status, education, and recreational physical activity were recorded at the baseline wave. Education was categorised as less than technical or high school, completed technical or high school, or completed a trade or tertiary degree or diploma. Smoking status was categorised as never-smoker, former-smoker, currently smoking 1–14 cigarettes per day and currently smoking 15 or more cigarettes per day at the time of the baseline exam. Country of birth was dichotomised into Northern European descent (Australia, New Zealand, England, Ireland, Scotland, Wales or Latvia) and Southern European Descent (Italy, Greece or Malta). Recreational physical activity during the 6 months prior to the baseline exam was categorised into quartile groupings.

Late AMD was defined as the presence of choroidal neovascularization or geographic atrophy in either eye [42]. If only one eye was graded, that participant was omitted from the analysis, unless late AMD was detected in that eye.

Vital status was obtained through probabilistic matching with the Australian National Death Index. Participants were considered to have missing data at follow-up if they survived until the start of the wave but had missing outcome data. The later stages of AMD are rarely seen in people less than 50 years of age, therefore participants who were projected to be less than 50 years of age at the start of the follow-up wave were excluded from this analysis.

Covariate balance was assessed among participants with non-missing outcome data before and after applying MSM weights by calculating the standardised difference between high and low iron take groupings [33]. Bias-corrected 95% confidence intervals were generated via 1500 bootstrap samples for each of the estimates [38]. Because it is assumed that the unmeasured confounders will have an opposing net effect on the probability of survival and the probability of AMD (i.e. variables that decrease the probability of survival will increase the probability of AMD), the sensitivity analysis was restricted to values of the sensitivity parameter equal to or greater than one.

Data generation and all statistical analyses were performed using Stata/SE version 14.2 (StataCorp LP, College Station, TX, USA) [43].

## Results

### Simulation study results

A total of 12 scenarios and 14,400 datasets were analysed.

#### Covariate balance

The predictors of exposure, sex and age, were unbalanced between iron intake levels (average standardised difference ≥ 21% and ≥ 26% for each scenario, respectively) for attending survivors, as seen in Additional file 1: Table S2. Balance was achieved after applying the MSM weight. However, a small difference after weighting remained among scenarios which were compliant with the monotonicity assumption (≤ 2.2% and ≤ 3.0%, respectively). Location of residence was a predictor of missingness (but not exposure) and was well balanced between iron intake levels across scenarios before and after weighting (≤ 0.8%). The distribution of genotype was not balanced across iron intake levels despite the exposure being generated independently of genotype. This imbalance was largest for scenarios generated with a negative effect of genotype on both survival and outcome (i.e., when *OR*_*UZ*_ = *OR*_*UY*_ = 0.50) and no violation of the monotonicity assumption. The standardised difference for genotype between exposure levels increased after applying the MSM weight for the majority of scenarios.

#### Empirical value of *τ*

The empirical value of *τ* (i.e. the ratio of the odds of AMD among the compliant-survivors to the odds of AMD among the always-survivors when *a* is set to one) was influenced by the values of *α*_*UZ*_ and *β*_*UY*_ and varied according to random sampling between simulated datasets.

The presence of defiant-survivors (in scenarios with violation of the monotonicity assumption) did not alter the value of *τ* which describes the difference in outcome distribution between always-survivors and compliant-survivors only.

In scenarios with a null effect of the unmeasured variable (*U*) on the outcome (meaning there is no survival bias), the average value of *τ* was 1.00 (see Additional file 1: Table S3).

**Fig. 3:**
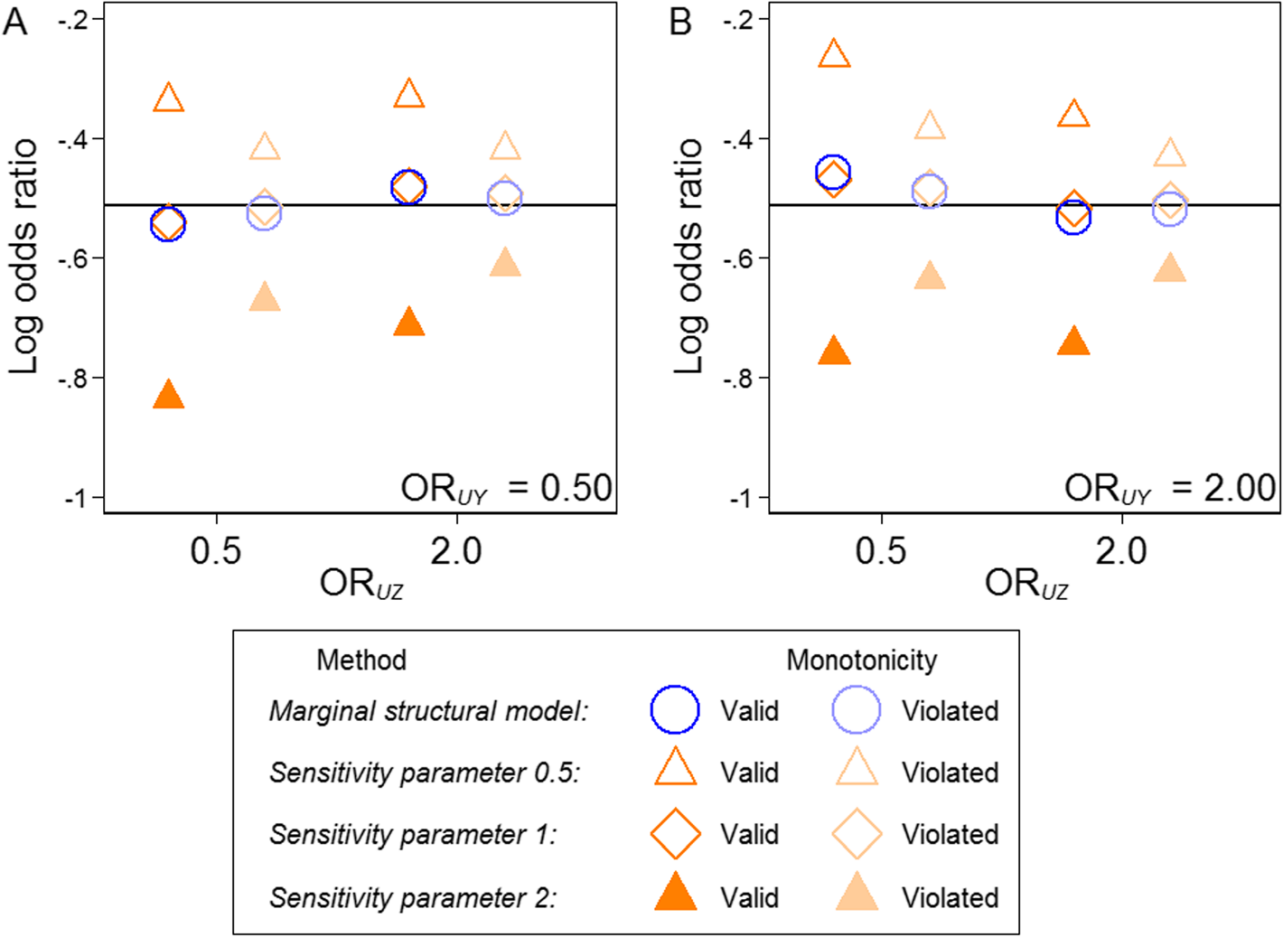
Estimates from the simulation study. Estimated using 10,000 observations simulated 1200 times for each scenario. The odds ratio effect of the unmeasured variable (*U*) on the outcome (*Y*), *OR*_UY_, was set to 0.5 in (**a)** and to 2 in (**b)**. The black line represents the true exposure effect (on the log odds ratio scale) of − 0.51. *OR*_UZ_ is the odds ratio of the unmeasured variable, *U*, on survival, *Z*

Across the remaining scenarios the average value of *τ* ranged between 0.89 and 1.12 (see Table 1) and were much less extreme than the sensitivity parameters of 0.5 and 2 chosen as proxies for *τ* when estimating the SACE via the sensitivity approach. Hence, the MSM estimates were more accurate than the sensitivity approach assessed at sensitivity parameters other than one in all scenarios (see Fig. 3).

**Table 1:** Log odds ratio estimates from simulation study

*OR*_*UY*_	*OR*_*UZ*_	Estimation method	Monotonicity									
			Valid					Violated				
			Estimate^a^	SE	SB	MSE	Coverage^b^	Estimate^a^	SE	SB	MSE	Coverage^b^
0.5	0.5	Average *τ*	0.92	0.09				0.92	0.09			
		Marginal structural model	−0.54	0.12	−29	0.01	91.6	− 0.53	0.11	−14	0.01	94.6
		Sensitivity analysis										
		SP = 0.5	−0.34	0.11	164	0.04		−0.42	0.10	92	0.02	
		SP = 1	−0.54	0.11	−26	0.01		−0.52	0.10	−4	0.01	
		SP = 2	−0.84	0.11	− 305	0.12		−0.67	0.10	− 159	0.04	
0.5	2.0	Average *τ*	1.10	0.11				1.10	0.11			
		Marginal structural model	−0.48	0.11	27	0.01	93.6	−0.50	0.10	11	0.01	92.4
		Sensitivity analysis										
		SP = 0.5	−0.33	0.10	176	0.04		−0.42	0.10	95	0.02	
		SP = 1	−0.48	0.10	30	0.01		−0.49	0.10	20	0.01	
		SP = 2	−0.71	0.10	− 197	0.05		−0.61	0.10	− 106	0.02	
2.0	0.5	Average *τ*	1.12	0.09				1.12	0.09			
		Marginal structural model	−0.46	0.10	54	0.01	90.4	−0.49	0.09	23	0.01	92.6
		Sensitivity analysis										
		SP = 0.5	−0.26	0.09	266	0.07		−0.38	0.09	143	0.02	
		SP = 1	−0.47	0.09	46	0.01		−0.48	0.09	33	0.01	
		SP = 2	−0.76	0.09	− 271	0.07		−0.64	0.09	− 140	0.02	
2.0	2.0	Average *τ*	0.90	0.08				0.89	0.07			
		Marginal structural model	−0.53	0.09	− 25	0.01	92.9	−0.52	0.08	−9	0.01	94.3
		Sensitivity analysis										
		SP = 0.5	−0.36	0.08	183	0.03		−0.43	0.08	104	0.01	
		SP = 1	−0.52	0.08	−7	0.01		−0.50	0.08	9	0.01	
		SP = 2	−0.75	0.08	− 290	0.06		−0.62	0.08	− 144	0.02	

^a^Estimates of the log odds ratio have been averaged over 1200 simulated datasets from each scenario

^b^Coverage indicates the percentage of datasets in each scenario where the true value of the SACE was within the bias-corrected bootstrap confidence interval of the marginal structural model

*MSE* Mean square error, *SACE* Survivor average causal effect, *SB* Standardized bias as a percentage, *SE* Empirical standard error, *SP* Sensitivity parameter.

*OR*_*UY*_ is the odds ratio effect of *U* on the outcome. *OR*_*UZ*_ is the odds ratio effect of U on survival. *τ* is the ratio of the odds of the outcome following high iron intake between compliant-survivors and always-survivors. True SACE log odds ratio = ln(0.6) = − 0.511.

#### Bias

Survival bias does not exist in the absence of an unmeasured confounder which acts as a shared predictor of survival and the outcome. When genotype was a predictor of survival but not the outcome, the estimated magnitude of bias was ≤0.005 (on the log odd ratio scale) for the MSM and ≤ 0.015 for the sensitivity analysis assessed at a sensitivity parameter of 1 (see Additional file 1: Table S3 and Figure S2).

For scenarios with an opposing direction of effect of genotype between survival and outcome, true value of *τ* was greater than one, meaning that always-survivors with high iron intake had lower odds of AMD than compliant-survivors with high iron intake. When it was assumed in the statistical analysis that there was no difference in outcome between principal strata (i.e. when the sensitivity parameter was set to 1 or the MSM approach was employed) the estimated effect of high iron intake compared to low iron intake among always-survivors was therefore diluted towards the null and the true value of the SACE was underestimated (bias ≤0.042 and ≤ 0.054 for sensitivity analysis and MSM respectively).

For scenarios with the same direction of effect of genotype between survival and outcome, the true value of *τ* was less than one. In these scenarios, the always-survivors with high iron intake had greater odds of AMD than compliant-survivors with high iron intake. Therefore, when the odds of the outcome was assumed to be equal between principal strata in the analysis, the true effect of the exposure on the outcome amongst always-survivors was overestimated (magnitude of bias ≤0.028 and ≤ 0.033 for the sensitivity approach assessed at the sensitivity parameter of 1 and for the MSM approach, respectively).

When estimating the effect of the exposure on the outcome via the MSM approach, survival is modelled equally among those with each level of the exposure. This reflects the data generating process of scenarios created with a violation of monotonicity and explains why bias was lower among these scenarios.

#### Distribution of estimates

The standard error was fairly consistent across estimation methods and scenarios, although standard errors were slightly greater, on average, for scenarios generated under the monotonicity assumption compared to scenarios with a violation of the monotonicity assumption. Among scenarios with a non-null effect of genotype on the outcome, the majority of estimates had high values of standardised bias due to the relatively small value of the standard error compared to the absolute bias and coverage was lower than the nominal 95% confidence interval for the MSM estimates (Table 1).

### Results from example dataset

#### Participants, survival and outcome

**Fig. 4:**
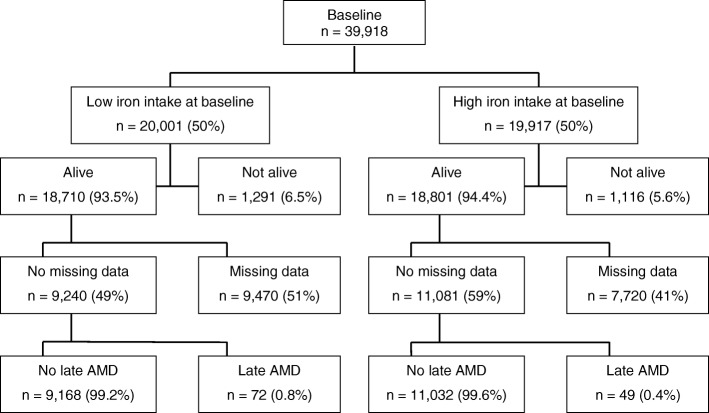
Flow chart of participants in the Melbourne Collaborative Cohort Study, 1990 to 2007. Participants who were alive at the start of the follow-up study wave but did not attend or had missing data on age-related macular degeneration (AMD) at follow-up were regarded to have missing data

Of the 39,918 participants recruited at baseline with complete data on the exposure and potential confounders, 37,511 (94%) survived until the start of the follow-up wave. Of those 20,321 (54%) had complete data on the outcome at the follow-up wave (Fig. 4). The covariate distribution was unbalanced between individuals with high and low iron intake (Table 2). The standardised difference decreased across all covariates after applying the MSM weighting scheme.

**Table 2:** Standardised difference between exposure groups for 20,321 participants of the Melbourne Collaborative Cohort Study with non-missing data on age-related macular degeneration status

	Iron Intake		Standardised difference	
	High	Low	Unweighted	Weighted
Mean age at follow-up (years)	64.1	64.0	−0.02	− 0.01
Sex				
Male	39.7	40.0	0.01	0.01
Female	60.3	60.0	−0.01	0.00
Smoking status (baseline)				
Never-smoker	57.1	63.4	0.13	0.00
Former-smoker	33.3	29.3	−0.09	−0.02
Current smoker				
Smoker 1–14 cigarettes/day	3.6	2.9	−0.04	−0.01
Smoker > 14 cigarettes/day	5.9	4.4	−0.07	0.03
Education (baseline)				
Less than high/technical school	52.1	44.4	−0.16	0.01
High/technical school	14.0	14.7	0.02	−0.01
Trade, tertiary degree or diploma	34.0	41.0	0.15	0.00
Country of birth				
Northern European	80.6	91.2	0.31	0.04
Southern European	19.4	8.8	−0.31	−0.04
Physical activity quartile (baseline)				
1 (Least active)	22.5	16.5	−0.15	−0.04
2	22.4	22.3	0.00	0.03
3	24.9	25.5	0.01	0.01
4 (Most active)	30.2	35.7	0.12	0.00

The marginal probability of survival following high and low iron intake after adjusting for baseline covariates was 94.1% (95% CI 94.1–94.2) and 93.8% (95% CI 93.7–93.8), respectively with an adjusted OR of 1.07 (95% CI 0.98–1.17).

Late AMD was detected in 121 (0.6%) of the participants who had data at the follow-up wave.

#### Survivor average causal effect

The estimated ORs and 95% CIs for the relationship between iron intake and late AMD are presented in Table 3, with all estimates suggesting a protective association between high dietary iron intake and the later stages of AMD. The estimates were similar for each of the five approaches, suggesting only minimal survival bias in this analysis.

**Table 3:** Association between iron intake and late age-related macular degeneration among 39,918 participants of the Melbourne Collaborative Cohort Study

Method^a^	OR	(95% CI)^b^
Complete case^c^	0.572	(0.396, 0.818)
	SACE OR	(95% CI)^b^
Marginal structural model	0.536	(0.368, 0.789)
Sensitivity analysis		
Sensitivity parameter = 1	0.583	(0.374, 0.780)
Sensitivity parameter = 2	0.581	(0.374, 0.777)

^a^ Each model adjusted for age, sex, country of birth, smoking status, physical activity and educational attainment

^b^ Bias corrected confidence intervals estimated via 1500 bootstrap samples

^c^ Naïve log-odds of AMD associated with iron intake estimated via complete case multivariable logistic regression analysis among all survivors

*OR* Odds ratio, *SACE* Survivor average causal effect, *95% CI* 95% Confidence Interval.

The SACE OR estimated via the sensitivity analysis approach was 0.58 (95% CI 0.37, 0.78) when evaluated at both values of the sensitivity parameter (1 and 2). This is due to the difference in the marginal probability of survival between iron intake levels being small.

## Discussion

This paper compares approaches to explore exposure-outcome relationships in studies impacted by death and attrition due to other causes. Unobserved data due to death is distinct from that due to non-attendance: the predictors of death and attrition may be different and the outcomes for the deceased are undefined, rather than missing.

Covariate balance was achieved across exposure levels for measured covariates in both the simulation study and illustrative example of this paper. However, balance was not achieved for an unmeasured and shared predictor of survival and the outcome in the simulation study. An example of a potential shared survival-AMD confounder is the complement factor H gene. The minor allele of the Y402H single nucleotide polymorphism has been associated with decreased survival and an increased risk of AMD [44]. Conversely, the *ε* 4 variant of the apolipoprotein E gene is known to be associated with a decreased risk of both survival and AMD [45]. In reality, there will be several unmeasured variables that combine to influence principal strata. Information on participant genotype may not be available to investigators and the role of epigenetics in AMD development is not yet fully understood. Investigators should therefore carefully consider whether all shared predictors of survival and the outcome are likely to be measured. In settings where data is not available for known survival-outcome confounders, a confounding function approach may be of use [46, 47]. Further work is required to assess the application of the confounding function to studies at risk of survival bias.

### Illustrative example

A protective association between iron intake and late AMD is counterintuitive given the previously reported negative relationship with red meat and previous findings of increased levels of iron in the retinas of individuals with AMD [48–50]. Iron intake is likely to be highly correlated with the intake of other nutrients which are likely to be confounders of the iron-AMD relationship. It is possible that the decreased rates of mortality and AMD observed among participants with high dietary iron intake may be reflective of a diet which is generally high in essential nutrients. Given that iron intake was dichotomised and other nutrients were not adjusted for, additional in-depth analyses should be carried out to explore the relationship between iron intake and AMD further. Only a small difference in survival was observed between those with high and low iron intake and therefore survival bias did not seem to be influential in this example.

### Limitations of these methods

Although the problem of missing data can be addressed in a number of ways, only MSMs were assessed in this simulation study as they have previously been applied to estimate the magnitude of exposure-outcome associations in the presence of survival bias. MSMs can only adjust for measured confounders [51]. These models cannot mitigate the bias attributable to associations between the outcome and the probability of non-attendance which is likely to be present in large scale epidemiological studies if all underlying reasons of non-attendance have not been captured [30].

The use of inverse-probability weighting in the presence of attrition due to death has been criticized in the past because it requires the outcomes of the deceased to be considered as missing, rather than undefined [52]. In turn, a pseudo-population of survivors is created (via upweighting of survivors to represent the deceased) from which inferences are drawn. These methods rely on the assumptions that survival can be manipulated, and that living participants are suitable representatives of the dead. However, in the illustrative example the estimates from the MSM were similar to those from the sensitivity analysis. In addition, MSMs provide a point estimate, which may be viewed more favourably by content matter experts than the range of plausible values produced by the sensitivity approach, especially when assumptions about principal stratification are questionable. Nevertheless, in the presence of unmeasured survival-outcome confounders, that point estimate may be biased. For this reason, it is important to highlight the potential for bias and speculate on the direction of this bias if these methods are applied.

Even when working closely with content experts, it can be difficult to ascertain which values of the sensitivity parameter to employ. While advice on eliciting information from subject matter experts for sensitivity parameters for handling missing not at random data is available, further work is required to guide the elicitation of plausible values for the sensitivity parameter used to deal with survival bias [53, 54].

The sensitivity approach is slightly more complex to compute than the MSM approaches. However, methods presented in this paper can be executed using standard statistical software.

## Conclusions

The direction and magnitude of survival bias are directly related to the direction and magnitude of effect of shared survival-outcome confounders. Therefore, it is essential that content experts and data analysts together prepare causal diagrams that include nodes for survival and hypothesised measured and unmeasured survival-outcome confounders to guide the selection of the analysis method. The SACE will be most useful when the exposure of interest is strongly associated with survival.

## Supplementary information

**Additional file 1.** Data generating mechanisms and Stata computing code for simulation study.

## Data Availability

The data analysed for the simulation portion of this study were generated using the computing code included in the Additional file 1 published with this article. Datasets from the Melbourne Collaborative Cohort Study are not publicly available due to privacy reasons.

## Abbreviations

95% CI: 95% confidence interval
AMD: Age-related macular degeneration
AS: Always-survivor
CS: Compliant-survivor
MSM: Marginal structural model
OR: Odds ratio
SACE: Survivor average causal effect
