# Correction to: A comparison of methods to estimate the survivor average causal effect in the presence of missing data: a simulation study

**DOI:** 10.1186/s12874-020-00935-x

**Published:** 2020-02-27

**Authors:** Myra B. McGuinness, Jessica Kasza, Amalia Karahalios, Robyn H. Guymer, Robert P. Finger, Julie A. Simpson

**Affiliations:** 1 Centre for Eye Research Australia, Royal Victorian Eye and Ear Hospital, Melbourne, Australia; 2 Centre for Epidemiology and Biostatistics, Melbourne School of Population and Global Health, University of Melbourne, Melbourne, Australia; 3 Department of Epidemiology and Preventive Medicine, Monash University, Melbourne, Victoria 3010 Australia; 4 Ophthalmology, Department of Surgery, University of Melbourne, Melbourne, Australia; 5 Department of Ophthalmology, University of Bonn, Bonn, Germany; 6 Cancer Epidemiology Centre, Cancer Council Victoria, Melbourne, Australia

**Correction to: BMC Med Res Methodol**

**https://doi.org/10.1186/s12874-019-0874-x**

In the original publication of this article [1], the incorrect causal diagram was submitted as Fig. 1. The figure published in the original article depicts an exposure measured at two study waves. The correct causal diagram is presented in two panels and represents the relationship between an exposure measured at a single study wave and the outcome. This correction does not impact the original figure legend or manuscript. The corrected Fig. 1 is shown below.

**Fig. 1:**
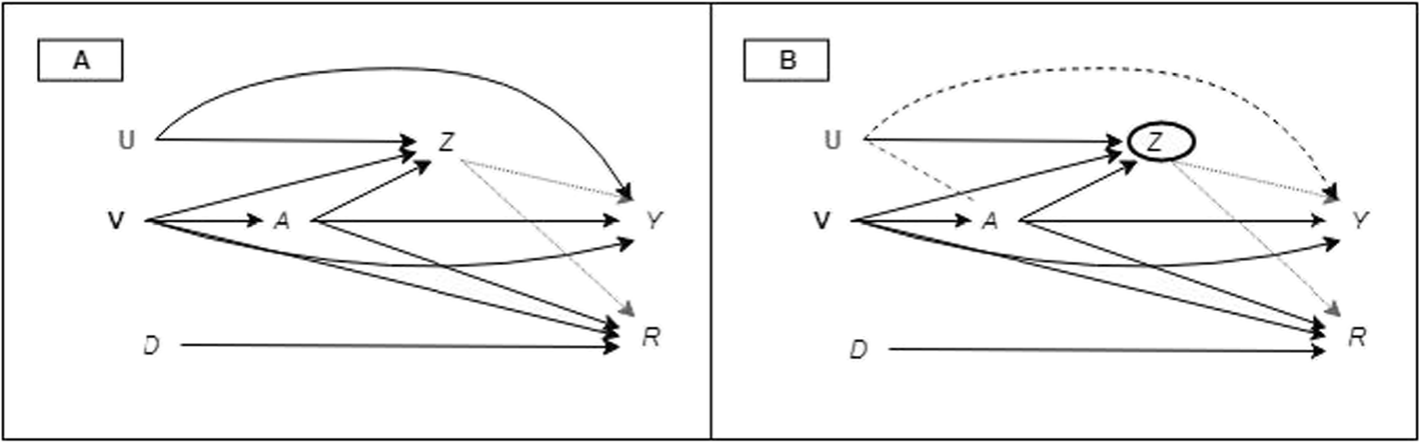
Causal diagram for the effect of iron intake on age-related macular degeneration. V represents the vector of participant demographics (e.g. age and sex) recorded at baseline. Exposure, A, is also recorded at baseline. Z is an indicator of survival until the start of the follow-up wave. R is an indicator of attendance at the follow-up study wave when outcome (Y, age-related macular degeneration) was ascertained. An indicator genotype, U, is unmeasured, as is D, an indicator for area of residence. **a** A scenario where missing outcome data are missing at random. **b** Conditioning on Z (a collider between the exposure and U) will unblock the backdoor pathway (dashed line) from the exposure to the outcome through U

## References

[1] McGuinness (2019). BMC Med Res Methodol.

