# Supplementary material for: A comparison of methods to estimate the survivor average causal effect in the presence of missing data: a simulation study
Source: BMC Med Res Methodol. 2019 Dec 3;19:223. doi: 10.1186/s12874-019-0874-x (PMC6892197; doi:10.1186/s12874-019-0874-x)
Supplement: Supplementary file 1 — Additional file 1. Data generating mechanisms and Stata computing code for simulation study. [file 12874_2019_874_MOESM1_ESM.docx]

**Additional File 1: Data generating mechanisms for simulation study**

**A Comparison of Methods to Estimate the Survivor Average Causal Effect in the Presence of Missing Data: a Simulation Study**

Myra B. McGuinness, MBiostat, Jessica Kasza, PhD, Amalia Karahalios, PhD, Robyn H. Guymer, MBBS, PhD, Robert P. Finger, MBBS, PhD, Julie A. Simpson, PhD

Data generation and all statistical analyses were performed using Stata/SE version 15.1 (StataCorp LP, College Station, TX, USA) with the Mersenne Twister random number generator and a starting seed of 13073199.[1]

For each scenario, a dataset of 10,000 observations was simulated 1,200 times. The interval between the baseline and follow-up wave was assumed to be equal for all participants. The program was written to disregard any dataset generated without sufficient cases of age-related macular degeneration (AMD) for analysis and loop until 1,200 valid datasets had been generated. However no datasets were disregarded during this simulation study.

The parameters used to generate the data are provided in Table 1 below.

Reference

1. Matsumoto M, Nishimura T: **Mersenne twister: a 623-dimensionally equidistributed uniform pseudo-random number generator**. *ACM Transactions on Modeling and Computer Simulation (TOMACS)* 1998, **8**(1):3-30.

Table S1: Parameters used to generate data for simulation study

| **Variable** | **Population model** |
| --- | --- |
| Sex $\left[ V1 \right]$  0 Male  1 Female | $V1\sim Bin(p=0.5)$ |
| Mean-centred age $\left[ V2 \right]$ | $V2\sim U(-14, 15)$ |
| Genotype $\left[ U \right]$  0 Absent  1 Present | $U\sim Bin(p=0.33)$ |
| Residence $\left[ D \right]$  0 High socioeconomic area  1 Low socioeconomic area | $D\sim Bin(p=0.5)$ |
| Iron intake $\left[ A \right]$  0 Low iron intake  1 High iron intake | $\mathrm{logit}\left( A \right)=\ln\left( 0.75 \right)V1+\ln\left( 0.95 \right)V2$ |
| Potential survival when $a=1$ $\left[ Z(a=1) \right]$  0 Deceased  1 Alive | $\mathrm{logit}\left( Z(a=1) \right)=\mathrm{logit}\left( z \right)+\ln\left( 2 \right)$ |
| Potential survival when $a=0$ $\left[ Z(a=0) \right]$  0 Deceased  1 Alive | If compliant with the monotonicity assumption:  $\mathrm{logit}\left( Z(a=0) \right)=\mathrm{logit}\left( z \right)$ if $Z\left( a=1 \right)=1$  If monotonicity assumption violated:  $\mathrm{logit}\left( Z(a=0) \right)=\mathrm{logit}\left( z \right)$ |
| Strata $\left[ S \right]$  0 Never-survivor  1 Always-survivor  2 Compliant-survivor  3 Defiant-survivor | $S=0$ if $Z\left( a=0 \right)=0$ & $Z\left( a=1 \right)=0$  $S=1$ if $Z\left( a=0 \right)=1$ & $Z\left( a=1 \right)=1$  $S=2$ if $Z\left( a=0 \right)=0$ & $Z\left( a=1 \right)=1$  $S=3$ if $Z\left( a=0 \right)=1$ & $Z\left( a=1 \right)=0$ |
| Survival status when $a=A$ $\left[ Z \right]$  0 Deceased  1 Alive | $Z=0$ if ($Z\left( a=0 \right)=0$ & $A=0$) or ($Z\left( a=1 \right)=0$ & $A=1$)  $Z=1$ if ($Z\left( a=0 \right)=1$ & $A=0$) or ($Z\left( a=1 \right)=1$ & $A=1$) |
| Potential outcome when $a=0$ $\left[ Y(a=0) \right]$  0 Disease absent  1 Disease present | $\mathrm{logit}\left( Y(a=0) \right)=\mathrm{logit}\left( y \right)$ if $Z\left( a=0 \right)=1$ |
| Potential outcome when $a=1$ $\left[ Y(a=1) \right]$  0 Disease absent  1 Disease present | $\mathrm{logit}\left( Y(a=1) \right)=\mathrm{logit}\left( y \right)+ln(0.6)$ if $Z\left( a=1 \right)=1$ |
| Non-missing outcome data $\left[ R \right]$  0 Missing outcome data  1 Outcome data not missing | $R=.$ if $Z=0$  $\mathrm{logit}\left( R \right)=\ln\left( 4 \right)+\ln\left( 2 \right)V1+\ln\left( 1.4 \right)A+ln(0.8)D$  if $Z=1$ |
| Age-related macular degeneration $\left[ Y \right]$  0 Absent  1 Present | $Y=Y(a=0)$ if $A=0$ & $Z=R=1$  $Y=Y\left( a=1 \right)$ if $A=1$ & $Z=R=1$  $Y= .$ if $Z= 0$ or $R= 0$ |
| Model coefficients: $\boldsymbol{\alpha}_{\mathbf{UZ}}=$ $\ln\left( 0.5 \right)$ or $ln(2)$; $\boldsymbol{\beta}_{\mathbf{UY}}=\ln\left( 0.5 \right)$, $ln(1)$ or $ln(2)$; $\boldsymbol{\gamma}_{\mathbf{V2Y}}=\ln\left( 0.95 \right)$ or $\ln\left( 1.05 \right)$  $\mathrm{logit}\left( z \right)=\ln\left( 1.5 \right)+\ln\left( 2 \right)V1+\ln(0.95)V2+\boldsymbol{\alpha}_{\mathbf{UZ}}U$  $\mathrm{logit}\left( y \right)=\ln\left( 0.11 \right)+\ln\left( 1.5 \right)V1+\ln\left( 1.05 \right)V2+\boldsymbol{\beta}_{\mathbf{UY}}U$ | |

Generate sex $[V1]$, mean-centred age $[V2]$, an indicator for poor health $\left[ U \right]$ and an indicator of area of residence $[D]$ for 10,000 participants.

Generate potential survival for high iron intake $[Z(a=1)]$ conditional on $V1$, $V2$ and $U$.

**For scenarios compliant with the monotonicity assumption:**

Generate potential survival for low iron intake $[Z(a=0)]$ conditional on $V1$, $V2$ and $U$ for participants who would survive following high iron intake (i.e. for those with $Z\left( a=1 \right)=1$)

**For scenarios that violate the monotonicity assumption:**

Generate potential survival under low iron intake $[Z(a=0)]$ conditional on $V1$, $V2$ and $U$ for all participants.

Derive observed survival status $[Z]$ according potential survival status $[Z(a=1)$ and $Z(a=1)]$ and exposure [$A]$

Generate potential outcomes for low iron intake $\left[ Y\left( a=0 \right) \right]$ conditional on $V1$, $V2$ and $U$ for participants who would survive under low iron intake (i.e. for those with $Z\left( a=0 \right)=1$)

Generate potential outcomes for high iron intake $\left[ Y\left( a=1 \right) \right]$ conditional on $V1$, $V2$and $U$ for participants who would survive under high iron intake (i.e. for those with $Z\left( a=1 \right)=1$)

Allocate observed outcome for age-related macular degeneration $[Y]$ deterministically according to $A$, $Z$, $R$ and potential outcome (i.e. $Y(a=0)$ or $Y(a=1))$

Generate the exposure, iron intake $[A]$, conditional on $V1$ and $V2$.

Generate an indicator of non-missing outcome data [$R]$ conditional on $V1$, $V2$ $D$ and $A$ for survivors (i.e. those with $Z=1$).

Allocate strata $[S]$ according to potential survival status $[Z(a=1)$ and $Z(a=1)]$

Figure S1: Flow chart of data generation process. Datasets were generated and analysed 1,200 times under each scenario

**Stata code for simulation study**

// Generate single dataset

capture program drop sim_iron_data

program define sim_iron_data, rclass

syntax, vm(numlist max = 1) uy(numlist max = 1) uz(numlist max = 1)

clear

quietly set obs 10000

tempvar pz z0 z1 strata py y0 y1

* Exposure and covariates

quietly gen U = uniform() < invlogit(ln(0.5)) // unmeasured genotype

quietly gen V1 = uniform() < invlogit(0) // sex

quietly gen V2 = floor(30 * runiform() - 15) // Mean centred age [-14,15]

quietly gen A = uniform() < invlogit((ln(0.75)*V1) + (ln(0.95)*V2)) // iron

quietly gen D = uniform() < invlogit(0) // residence

* Survival

quietly gen Z = .

quietly gen `pz' = ln(1.5) + (ln(2) * V1) + (ln(0.95) * V2) + (ln(`uz') *U)

quietly gen `z1' = uniform() < invlogit(`pz' + ln(2))

quietly gen `z0' = 0

if `vm' == 0 {

quietly replace `z0' = uniform() < invlogit(`pz') if `z1' == 1

}

if `vm' == 1 { // Violation of monotonicity

quietly replace `z0' = uniform() < invlogit(`pz')

}

foreach b of numlist 0/1 {

quietly replace Z = `z`b'' if A == `b'

}

* Strata

quietly gen `strata' = 2 // compliant-survivors

quietly replace `strata' = 0 if `z0' == 0 & `z1' == 0 // never-survivors

quietly replace `strata' = 1 if `z0' == 1 & `z1' == 1 // always-survivors

quietly replace `strata' = 3 if `z0' == 1 & `z1' == 0 // defiant-survivors

* Outcome

quietly gen Y = .

quietly gen `py' = ln(0.11) + (ln(`uy') * U) + (ln(1.5) * V1)+(ln(1.05)*V2)

quietly gen `y0' = uniform() < invlogit(`py') if `z0' == 1

quietly gen `y1' = uniform() < invlogit(`py' + ln(0.6)) if `z1' == 1

foreach b of numlist 0/1 {

quietly replace Y = `y`b'' if A == `b'

}

* Empirical value of tau

quietly logistic `y1' i.`strata' i.(V1) V2 if inlist(`strata',1,2)

return scalar tau = exp(_b[2.`strata'])

* Non-missing data

quietly gen R = .

quietly replace R = uniform() < invlogit(ln(4) + (ln(1.4) * A) + ///

(ln(2) * V1) + (ln(0.9) * V2) + (ln(0.8) * D)) if Z == 1

quietly replace Y = . if R == 0

end

// Analyse single dataset

capture program drop analyse_sace_iron

program define analyse_sace_iron, eclass

version 14

preserve

tempvar ax sigmax as ar ssw g0 g1 h0 h1 gh0 gh1

tempname mat

* Propensity for exposure

quietly logistic A i.V1 V2

quietly predict `ax'

sum `ax' if A == 1, meanonly

quietly gen `sigmax' = r(mean)

sum `ax' if A == 0, meanonly

quietly replace `sigmax' = r(mean) if e(sample)

quietly replace `ax' = 1 - `ax' if A == 0

* Propensity for survival

quietly logistic Z i.(A V1) V2

quietly predict `as'

foreach i of numlist 0 1 {

quietly logistic Z i.V1 V2 if A == `i'

quietly predict `g`i''

}

* Propensity for non-missing data

quietly logistic R i.(A V1) V2

quietly predict `ar' if e(sample)

* Generate weights

quietly gen `ssw' = (`sigmax') /(`ax' * `as' * `ar')

* Marginal structural models

quietly logistic Y i.(A V1) V2 [pweight = `ssw']

local essw = _b[1.A]

* Sensitivity approach

foreach b of numlist 0/1 {

sum `g`b'', meanonly

local nu_`b' = r(mean) // marginal probability of survival

quietly logistic Y i.V1 V2 if A == `b'

quietly predict `h`b''

quietly gen `gh`b'' = `g`b'' * `h`b''

sum `gh`b'' if Z == 1, meanonly

local xi_`b' = r(mean) // marginal probability of both survival and AMD

}

local sa1 = ln((`xi_1') * (`nu_0'-`xi_0') / (`xi_0' * (`nu_1'-xi_1')))

foreach t of numlist 0.5 2 { // sensitivity parameters

if `t' == 2 {

local ta = 2

}

else {

local ta = 0

}

local q_`ta' = sqrt(((`nu_0' + `xi_1') * (1 - `t') + (`t' * `nu_1'))^2 ///

+ (4 * `xi_1' * `nu_0' * (`t' - 1)))

local sa`ta' = ln(((`nu_0' - `xi_0') / `xi_0') * ///

((`nu_0' + `xi_1') * (`t' - 1) - (`t' * `nu_1') + `q_`ta'') / ///

((`nu_0' - `xi_1') * (`t' - 1) + (`t' * `nu_1') - `q_`ta''))

}

* Covariate balance

quietly xi: pbalchk A V1 V2 U D if Y != ., nocatstandardize

matrix CBu = r(ssmeandiff) // unweighted

quietly xi: pbalchk A V1 V2 U D if Y != ., wt(`ssw') nocatstandardize

matrix CB`ssw' = r(ssmeandiff) // weighted

* Output

matrix `mat' = `essw', `sa0', `sa1', `sa2', CBu, CBssw

ereturn post `mat'

restore

end

Table S2: Average standardised difference in covariate levels between exposure groups among attending survivors, generated with 1,200 repetitions per scenario.

| **Scenario**  **number** | **Violation**  **of**  **monotonicity** | **Effect of genotype** | | **Covariate** | | | | | | | |
| --- | --- | --- | --- | --- | --- | --- | --- | --- | --- | --- | --- |
|  |  | **Survival** | **Outcome** | **Sex** | | **Age** | | **Area of residence** | | **Unmeasured genotype** | |
|  |  | ${OR}_{UZ}$ | ${OR}_{\boldsymbol{UY}}$ | **Unweighted** | **Weighted** | **Unweighted** | **Weighted** | **Unweighted** | **Weighted** | **Unweighted** | **Weighted** |
| 1 | No | 0.5 | 0.5 | -0.28 | -0.02 | -0.26 | 0.03 | 0.01 | 0.00 | 0.11 | 0.12 |
| 2 | No | 0.5 | 1.0 | -0.28 | -0.02 | -0.26 | 0.03 | 0.00 | 0.00 | 0.11 | 0.12 |
| 3 | No | 0.5 | 2.0 | -0.28 | -0.02 | -0.26 | 0.03 | 0.01 | 0.00 | 0.11 | 0.12 |
| 4 | No | 2.0 | 0.5 | -0.25 | -0.01 | -0.29 | 0.02 | 0.01 | 0.00 | -0.08 | -0.09 |
| 5 | No | 2.0 | 1.0 | -0.25 | -0.01 | -0.30 | 0.01 | 0.01 | 0.01 | -0.08 | -0.09 |
| 6 | No | 2.0 | 2.0 | -0.25 | -0.01 | -0.30 | 0.01 | 0.01 | 0.00 | -0.08 | -0.09 |
| 7 | Yes | 0.5 | 0.5 | -0.21 | 0.00 | -0.34 | 0.00 | 0.01 | 0.00 | 0.05 | 0.04 |
| 8 | Yes | 0.5 | 1.0 | -0.21 | 0.00 | -0.34 | 0.00 | 0.01 | 0.01 | 0.05 | 0.04 |
| 9 | Yes | 0.5 | 2.0 | -0.21 | 0.00 | -0.34 | 0.00 | 0.01 | 0.01 | 0.05 | 0.04 |
| 10 | Yes | 2.0 | 0.5 | -0.21 | 0.00 | -0.35 | 0.00 | 0.01 | 0.01 | -0.04 | -0.04 |
| 11 | Yes | 2.0 | 1.0 | -0.21 | 0.00 | -0.35 | 0.00 | 0.01 | 0.00 | -0.04 | -0.04 |
| 12 | Yes | 2.0 | 2.0 | -0.21 | 0.00 | -0.35 | 0.00 | 0.01 | 0.00 | -0.04 | -0.04 |
| ${OR}_{UZ}$ = odds ratio effect of unmeasured genotype on survival; ${OR}_{UY}$ = odds ratio effect of unmeasured genotype on outcome | | | | | | | | | | | |

Table S3: Log odds ratio estimates from simulation study scenarios with a null effect of the unmeasured variable on the outcome.

| ${OR}_{UY}$ | ${OR}_{UZ}$ | **Estimation method** | **Monotonicity** | | | | | | | |
| --- | --- | --- | --- | --- | --- | --- | --- | --- | --- | --- |
|  |  |  | **Valid** | | | | **Violated** | | | |
|  |  |  | **Estimate*** | **SE** | **SB (%)** | **MSE** | **Estimate*** | **SE** | **SB (%)** | **MSE** |
| 1.0 | 0.5 | Average $\tau$ | 1.00 | 0.10 |  |  | 1.00 | 0.09 |  |  |
|  |  | Marginal structural model | -0.51 | 0.11 | -3 | 0.01 | -0.52 | 0.10 | -4 | 0.01 |
|  |  | Sensitivity analysis |  |  |  |  |  |  |  |  |
|  |  | SP = 0.5 | -0.31 | 0.10 | 195 | 0.05 | -0.41 | 0.10 | 106 | 0.02 |
|  |  | SP = 1 | -0.52 | 0.10 | -5 | 0.01 | -0.51 | 0.10 | 6 | 0.01 |
|  |  | SP = 2 | -0.81 | 0.10 | -296 | 0.10 | -0.66 | 0.10 | -157 | 0.03 |
| 1.0 | 2.0 | Average $\tau$ | 1.00 | 0.09 |  |  | 1.00 | 0.10 |  |  |
|  |  | Marginal structural model | -0.51 | 0.10 | -1 | 0.01 | -0.51 | 0.10 | 3 | 0.01 |
|  |  | Sensitivity analysis |  |  |  |  |  |  |  |  |
|  |  | SP = 0.5 | -0.35 | 0.09 | 173 | 0.03 | -0.42 | 0.09 | 99 | 0.02 |
|  |  | SP = 1 | -0.50 | 0.09 | 9 | 0.01 | -0.50 | 0.09 | 17 | 0.01 |
|  |  | SP = 2 | -0.73 | 0.09 | -242 | 0.06 | -0.62 | 0.09 | -117 | 0.02 |
| * Estimates of the log odds ratio have been averaged over 1,200 simulated datasets from each scenario.  MSE = mean square error; SACE = survivor average causal effect; SB = standardized bias as a percentage; SE = empirical standard error; SP = sensitivity parameter.  ${OR}_{UY}$ is the odds ratio effect of *U* on the outcome. ${OR}_{UZ}$ is the odds ratio effect of U on survival. $\tau$ is the ratio of the odds of the outcome following high iron intake between compliant-survivors and always-survivors. True SACE log odds ratio = ln(0.6) = -0.511 | | | | | | | | | | |


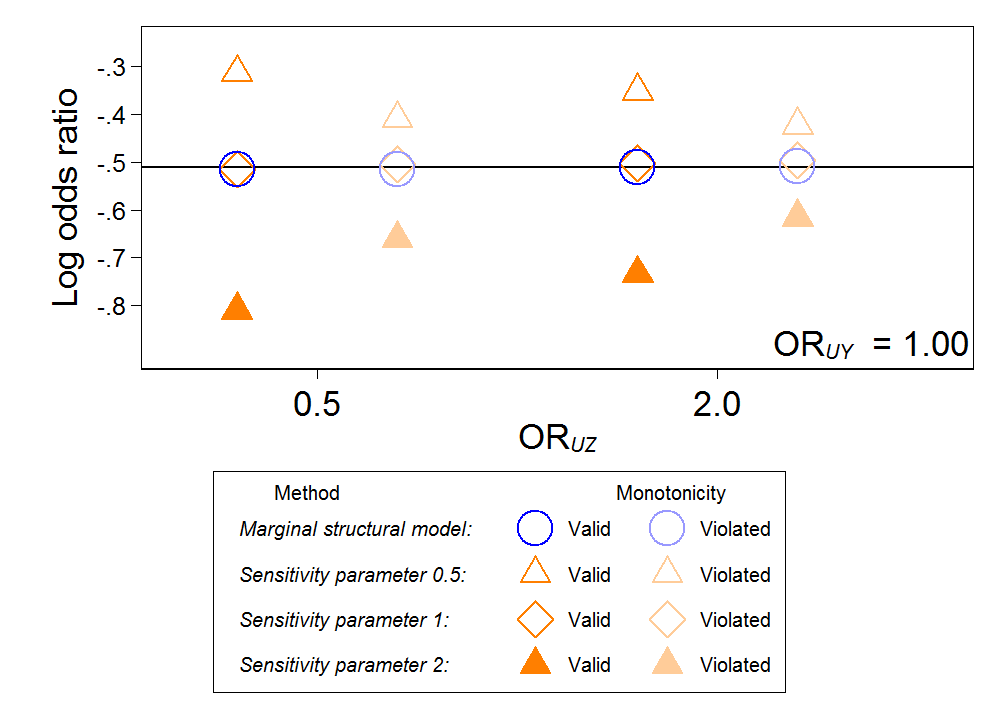


Figure S2: Estimates from simulation study scenarios with a null effect of the unmeasured variable on the outcome. Estimated using 10,000 observations simulated 1,200 times for each scenario. The odds ratio effect of the unmeasured variable $\left( U \right)$ on the outcome $\left( Y \right)$, ${OR}_{\text{UY}}$, was set to 1.0. The black line represents the true exposure effect (on the log odds ratio scale) of -0.51. ${OR}_{\text{UZ}}$ is the odds ratio of the unmeasured variable, $U$, on survival, $Z$.

**Proof for Equation 12**

The notation for the following equations is as defined in the manuscript. Measured exposure-outcome confounders are denoted $\mathbf{V}$.

Note, in the illustrative example, the exposure level given by $A=1$ (high iron intake) is considered to be predictive of greater levels of survival and a lower probability of an undesirable outcome (age-related macular degeneration). Whereas the in the paper by Egleston (2007) referred to below, an exposure level equal to one (indicating vision loss) is hypothesised to be associated with lower rates of survival and a greater risk of the undesirable outcome (emotional distress). Therefore, there are differences in the notation between the two papers.

Under the assumptions of ignorable treatment assignment (conditional on measured confounders) and outcome values missing at random:

| $\Pr\left[ Z\left( a \right)=1 \right]$ |  |  |
| --- | --- | --- |
| $=\Pr\left[ Z=1\vert\mathbf{V},A=a \right]=\nu_{a}$ | (From Egleston 2007, page 537) | (1) |

| $\Pr[Y\left( a \right)=1,Z\left( a \right)=1\vert\mathbf{V}]$ |  |  |
| --- | --- | --- |
| $=\Pr[Y=1,Z=1\vert\mathbf{V},A=a]=\xi_{a}$ | (From Egleston 2007, page 537) | (2) |

Under the assumptions of conditional ignorability, and outcome values missing at random:

| $\Pr\left[ Y\left( a=0 \right)=1 \vert\mathbf{V},AS \right]$ |  |  |
| --- | --- | --- |
| $=\Pr\left[ Y=1 \vert\mathbf{V},A=0,Z=1 \right]$ |  |  |
| $=\frac{\xi_{0}}{\nu_{0}}$ | (From Equation 4.1, Egleston 2007, page 533) | (3) |

Under the monotonicity assumption, this value is observable because all survivors with exposure $A=0$ are considered to be always-survivors.

| $\tau=\frac{\text{odds}\left[ Y(a=1)=1 \vert\boldsymbol{V},CS,Z=1 \right]}{\text{odds}\left[ Y(a=1)=1 \vert\boldsymbol{V},AS \right]}$ | (From Equation 4.8, Egleston 2007, page 536) | (4) |
| --- | --- | --- |

$\therefore$ when $\tau=1$:

| $\Pr\left[ Y\left( a=1 \right)=1 \vert\mathbf{V},CS,Z=1 \right]$ |  |  |
| --- | --- | --- |
| $=\Pr\left[ Y\left( a=1 \right)=1 \vert\mathbf{V},AS \right]$ |  |  |
| $=\Pr\left[ Y=1 \vert\mathbf{V},A=1,Z=1 \right]$ |  |  |
| $=\frac{\xi_{1}}{\nu_{1}}$ | (From Egleston 2007, page 536) | (5) |

Under the assumption of monotonicity, this value is also observable because all survivors with exposure $A=1$ will have the same distribution of $Y$, regardless of stratum.

| ${SACE}_{OR}(\tau)=\frac{\text{odds}\left[ Y(a=1)=1 \vert\boldsymbol{V},AS \right]}{\text{odds}\left[ Y(a=0)=1 \vert\boldsymbol{V},AS \right]}$ | (From Equation 3.1, Egleston 2007, page 530) | (6) |
| --- | --- | --- |

$\therefore$ when $\tau=1$:

| ${SACE}_{OR}(\tau=1)$ |  |  |
| --- | --- | --- |
| $=\frac{odds\left[ {\xi_{1}}/{\nu_{1}} \right]}{odds\left[ {\xi_{0}}/{\nu_{0}} \right]}$ |  |  |
| $=\frac{\xi_{1}(\nu_{0}-\xi_{0})}{\xi_{0}(\nu_{1}-\xi_{1})}$ | (Equation 12) | (7) |
